# Stories of Identity from High Performance Male Boxers in Their Training and Competition Environments

**DOI:** 10.3390/jfmk3040058

**Published:** 2018-11-24

**Authors:** Thierry R. F. Middleton, Jacob Dupuis-Latour, Yang Ge, Robert J. Schinke, Amy T. Blodgett, Diana Coholic, Brennan Petersen

**Affiliations:** 1 Human Studies Program, Laurentian University, 935 Ramsey Lake Road, Sudbury, ON P3E 2C6, Canada; 2 School of Human Kinetics, Laurentian University, 935 Ramsey Lake Road, Sudbury, ON P3E 2C6, Canada; 3 School of Social Work, Laurentian University, 935 Ramsey Lake Road, Sudbury, ON P3E 2C6, Canada

**Keywords:** empowering contexts, athletic identity, culture, elite athletes, storytelling

## Abstract

The current submission was conceived to broaden the discussion around male athletic identities by exploring the stories told by four members of the Canadian National Boxing Team. The athletes’ stories were elicited through an arts-based method followed by a conversational interview. Stories were then analyzed using an interpretive thematic analysis. Three salient themes were found—fluid masculinity, ethnicity brings an edge to boxing, and expressing identity through language. These themes present accounts that highlight how socially, culturally, and historically dominant narratives can allow athletes to feel comfortable in presenting the identities they might reveal or feel constrained from doing so due to factors outside of their control. The need to develop training and competition contexts that allow for the empowerment of athletes’ individually distinct identities is highlighted as a method to ensuring the positive mental health of elite level athletes.

## 1. Introduction

An increased focus on the mental health of athletes has led to sport psychology researchers emphasizing the need for athletes to be seen holistically, including their personal lives outside of sport [1,2]. Researchers have begun to explore the intersection of athletic identity with other identity constructs such as motherhood [3], racial identity [4], and physical disability [5]. Douglas, a former professional golf champion and qualitative researcher, has advocated for researchers to understand athletes’ lives through the stories that they tell [6]. This approach allows athletes’ identities to be understood beyond a narrow sport and performance focus, and rather as multiple, fluid, and ever-changing, impacted by the interactions individuals encounter and the cultural meanings given to these interactions [2]. Fundamental to this holistic athlete approach is considering the extent to which athletes feel comfortable in expressing themselves within their sporting environments [1]. Ronkainen, Watkins, and Ryba found that athletes felt that certain identities and practices were constrained due to the (re)produced and normalized understandings of gender, age, and the athletic body [7]. These constraints were impactful on athletes’ well-being and career decisions, with some athletes experiencing psychological distress and loneliness, leading some to terminate their elite careers at a young age.

Boxing is a sport that has historically encouraged and discouraged different forms of identities [1,8]. One dominant identity is that of the “ideal masculine image” characterized by violence and aggression that comes along with participating in a combative sport [9] As Woodward stated, boxing is historically a site where “there is hegemonic status afforded to heterosexual masculinity and the subordination of other masculinities, especially those constructed as weak or fearful and lacking courage” [10] (p. 8). This hegemonic masculinity is dependent on binary oppositions, with boxers having to negotiate a variety of dualistic positions such as big/small, strong/weak, and so on [10]. This conceptualization of masculinity is perhaps best summed up by Paradis who provides a definition of a “legitimate boxer as fearless, strong, courageous; someone with heart; the alpha male” [11] (p. 85).

Along with masculinity, boxing also used to be a space dominated by white Caucasian males; however, this began to change late in the nineteenth century as boxing fights became a venue to which all fans were drawn regardless of ethnicity or race [9]. Mixing of ethnicity and race in the gym has also become more common; for boxers at a gym in the United Kingdom that had predominantly been white, race and ethnicity were not considered a factor as boxers had joined the gym based on the reputation of the trainer [10]. However, having a diverse ethnic and racial membership does not come without complications. Previous work conducted within cultural sport psychology (CSP) has revealed the need for research and practice to open up space to include marginalized cultural identities [12]. This may be especially relevant to the members of the Canadian National Boxing Team as the team’s composition is immensely multicultural [13]. Stories told by female boxers of the Canadian National Boxing Team revealed how cultural differences impacted the expression of different identities within the boxing context [14]. The authors presented the analysis of the intersection of these boxers’ identities through the different lenses of a kaleidoscope, an impactful form of imagery that unveiled the fluid and dynamic nature by which the boxers negotiated their identities.

We aim to build upon this previous research by presenting a snapshot of different identities found within the stories told by male members of the Canadian National Boxing Team. Our aim is to bring greater focus to each identity, showing each as one lens into understanding the holistic identity of an elite level athlete. To further understand how male boxers described the different components of their identity, how they felt these were situated within the boxing context, and the ways in which this layered into their performance, this study was guided by the following two questions:

(1) How do male boxers on the Canadian National Boxing Team describe their identities?
(2) How do experiences related to these identities contribute to athletes’ sport performance?

## 2. Methodology

The current manuscript is part of a larger Social Sciences and Humanities Research Council (SSHRC) funded project led by the fourth author focused on the multifaceted identities of the male and female boxers representing the Canadian National Boxing Team. The project was borne out of prolonged contact between the fourth author and Boxing Canada, through his work as a mental performance consultant with the organization for the past 21 years. Recognized as the governing body for the sport of boxing in Canada by the Canadian Olympic Committee, Boxing Canada had great interest in understanding how a more inclusive, athlete-centered training environment and team structure could be developed. Recognizing that for an environment to become athlete-centered required that the athletes have their voices heard, it was decided an interpretive thematic analysis would be an appropriate approach. An interpretive thematic analysis enabled us to inductively analyze how male boxers described their identities and how they felt these were encouraged and/or discouraged in the national team environment [15].

### 2.1. Participants

Following research ethics board approval from the authors’ institution, emails were sent by Boxing Canada and the national sport organization’s high-performance director to the national team boxers to introduce the project. Interested boxers were asked to contact the project’s research coordinator to receive more information about the project, complete a consent form, and schedule an interview time. Both male and female boxers were recruited for this project; however, due to boxing being a historically male dominated sport we decided to stratify our sample by gender [10]. This narrow focus allowed us to focus this submission on developing a more in-depth and interpretive understanding of the stories told by male boxers. With our focus on specific knowledge associated with being a member of the male Canadian boxing team, we also decided to recruit only boxers who were mainstays on the team. Accordingly, our participants were four male boxers ranging in age from 20 to 34 years old (*M* = 24.75), each with multiple years of membership on the national team. Each boxer fought within a different weight category and had attended either the Olympics, Commonwealth games, and/or other major international competitions. Additionally, between the boxers they had won three major games medals and 13 national titles, and two had been ranked among the top 10 in the world. The four boxers also represented the multi-cultural composition of the National Boxing Team with two being immigrants, one a first generation Canadian, and one whose family had lived in Canada for several generations.

### 2.2. Situating the Researchers

For good quality thematic analysis, it is essential that the researchers play an active role throughout the research process [15]. Recognizing that this may play a role in the way the data is collected and analyzed, a brief introduction of authors backgrounds is vital [16]. The first author is a former elite level swimmer who is an immigrant to Canada and speaks multiple languages. The second author is a former elite level runner who grew up in a Francophone home, but now studies in English. Both authors are male and of Caucasian descent. The third author is an international student from China studying in Canada. She has consulting experience with her local boxing club, headed by one of the national boxing team coaches. She has also accompanied the fourth author to meetings with Boxing Canada’s executive board to present findings stemming from the funded project. As previously mentioned, the fourth author has over 20 years of consulting experience with both elite and professional boxers. He is also an experienced qualitative researcher, having conducted extensive research with a wide range of elite and multicultural athletes. The fifth author has extensive experience with conducting arts-based conversational interviews with multi-cultural athletes. Following our aim of eliciting strong dialogue between researcher and participants, she conducted all the interviews and provided continuous feedback during the analysis stage. The sixth author also has extensive experience with arts-based conversational interviewing. Her experience was integral to understanding how to centralize the athletes within the research process. The final author is a graduate student that was asked to provide critical feedback and further interpretation during the analysis and writing process. His previous research experience with team roles and role perceptions added another layer of knowledge into the interpretive process.

### 2.3. Data Collection

Owing to the vast physical distance between each athlete given that this national team was dispersed across the country, interviews were conducted using Skype, allowing for face-to-face contact. “Computer-mediated interviewing” comes with the benefit of physical distance between the interviewer and the participant, perhaps allowing some participants to feel more at ease with the researcher and permitting them to reveal information that may not be shared during an in-person interview [17]. However, for some participants this physical distance may also make an interview more impersonal and result in a lack of depth provided to answers [17]. To overcome these challenges, an arts-based method was used to open each interview helping to build a sense of comfort and rapport among the participant and researcher [18]. Additionally, arts-based methods have been used by researchers from various fields to help people draw on multiple senses and express their identities in a way that may not emerge otherwise [18]. To begin this process, athletes were asked to illustrate their identities as boxers by way of drawing a mandala (i.e., a drawing in a circle), a tree, or a river. Athletes who struggled with knowing where to begin were encouraged to engage in doodling as a warm-up exercise. For some, the anxiety at being “bad at art” was an obstacle, but they were reminded that their art piece was not going to be judged, but rather help them organize their thoughts and express themselves [18]. Once completed, participants photographed their drawing using their phone and emailed it to the interviewer. Participants who were unable to take a photo and/or share a picture of their drawing showed it to the interviewer via Skype video. Interpretation of the art work was done by the boxers through use of a conversational interview following the creation of their drawing [18].

Interviews were initiated through an open-ended question that related back to the art work: “Can you tell me about what you have drawn, in terms of who you are as an athlete?” and continued as a conversation. The interviews were conducted in either English or French depending on each boxer’s preference. Athletes were encouraged to take control of the conversational interview by sharing what they had chosen to include in their drawing, helping to centralize the aspects of themselves that they wished to share [19]. Further probing questions were asked related to the different identities and elements included in the athlete’s drawing, what they found comfortable or uncomfortable about being a national team boxer, and how these aspects of their identity were related to their performance in the ring. The interviews varied in length from 60 to 90 min, were recorded, and transcribed verbatim. Transcripts were then sent to the athletes for member reflections [20]. This was done for two reasons: (a) To allow athletes to reflect on the experiences they shared and add or change any of the shared information; and (b) to conduct research from a position of mutual respect and connectedness between researchers and participants. Allowing participants input into their shared experiences has been suggested as one way of working from a “culturally responsive relational reflexive position” [20] (p. 9). All interviews conducted in French were translated into English after this step.

### 2.4. Data Analysis

An interpretive thematic analysis was used to analyze the data [15]. The second and third authors immersed themselves in the data by first transcribing the recorded interviews verbatim and then reading the transcripts multiple times. During this reading, initial impressions of the identities expressed by athletes and the impact of these on competition were made via notes in the margins. Second, the notes were developed into initial codes that captured the contextual meanings of athletes’ identity experiences. Third, higher-order themes were developed from identified patterns across the initial codes that related back to boxers’ self-descriptions of their identities and how these identities impacted performance. Fourth, all authors reviewed the identified themes to ensure that they encapsulated the breadth of the coded data and that the codes within each theme highlighted the uniqueness of each facet of the athletes’ identity. Fifth, each theme was given a name that captured the essence of the identity focused on within that theme. Finally, narratives were developed that showcased how each identity was described by the athletes’, how they felt this identity was positioned in the boxing context, and the corresponding impact on performance. Quotations that provided vivid examples of the identity were nested within our narrative.

To ensure a high quality thematic analysis was produced, Braun and Clarke’s criteria were used to guide our research process [21]. The process began with a thorough transcription process, that also served as the initial familiarization with data. The process of coding the data, using these codes to develop themes, checking that the themes encapsulated the entirety of the data, and finally naming each theme was done in an iterative process to ensure that each data item was given equal attention, no data was missed during any stage, and that the extracts which were selected reflected the analytic claims we were making. Throughout these stages, and into the final stage of writing our narrative we aimed to craft a well organized story that remained in line with our research questions. Finally, by situating the researchers within the process we have highlighted our active role throughout the research process, rather than staking claim to providing an entirely “objective” account.

## 3. Results

The following section presents three themes that were salient through the athletes’ stories in relation to the way they constructed their identities. Theme one is centered on the fluid nature of how these boxers conceptualized masculinity, while theme two addresses how being proud of one’s ethnic background can have a positive effect on a boxer’s feelings of empowerment in the ring. Finally, theme three presents the role of language as a powerful force that brings together and/or divides athletes from one another. Pseudonyms are used throughout to ensure the boxers’ anonymity.

### 3.1. Fluid Masculinity

The boxers’ stories revealed masculinity to be an identity construct at a crossroads within the boxing environment. While the boxers spoke about a willingness to share alternative masculine views, an underlying message was that that there remains a dominant narrative associated with masculinity. Presented through two sub-themes, *Everyone has their own style and The paradox of looking good,* the tension between these two accounts was one bridged by the boxers’ talent and success.

#### 3.1.1. Everyone has Their Own Style

The relationship between how a boxer identified himself as a man and the way in which they fought in the ring was portrayed as unique to each boxer. More important than what style each person had was how they used their style when competing against others in the ring:

My personality as a boxer and as a man means I’m strong and agile. [Interviewer: Like that’s what you’re supposed to be as a male boxer?] No, no, no, no, no. Depending on the style. That’s what I’m saying, it depends on the style… Some guys are not as fast, and they’re not as agile, they’re not equivalent, they’re not as powerful at the same time as being all those things [i.e., athletic, strength, agility] together. That means it’s a different story for each person, and that’s just who I am… You have to use your style against their [other male athletes] style and you gotta make sure you flip the switch depending on the style, trying to fight the fight that you need to do- to beat that different opponent in the other corner. So that’s part of being in that gender.

(Ethan)

Ethan’s rich narrative involving the presence of different masculine styles inclusive of multiple characteristics (e.g., strength, agility, power) provides insight into what he conceptualizes as masculinity. His strong reaction (i.e., “No, no, no, no, no.”) to the interviewer’s question about the expectations of a narrow view of masculinity indicates that he is aware of the stereotypes that exist for male boxers but does not believe that these are warranted. This broadening view of masculinity and the accepted norm within boxing is portrayed as one under development, with boxers who did not fit the traditional mold needing to be successful to be accepted:

I mean I think you look better when you’re more masculine. But, really it doesn’t matter. As long as you’re a good boxer you don’t have to be skinny or big or fat. As long as you can box and be good that’s all that matters. [Interviewer: So basically if you’re getting the results, it doesn’t matter.] Yeah, just results.

(Oliver)

Oliver’s opinion on the importance of masculinity draws in the physical appearance aspects of how masculine boxers should appear. While his initial description of appearance as a factor that is not necessarily paramount to masculinity, and that the ideal appearance of male boxers is vast and fluid, he concludes by explaining that differing from the stereotypical appearance of a boxer is easier for those who are successful. Less experienced and/or less talented boxers may experience increased exclusion if their body composition and/or behaviors did not fit the muscular stereotypical image of a boxer. The portrayal of a more open and accepting sport is one heavily contingent on the success of those wishing to be different to the dominant view of masculinity within boxing, but does lead to an indication that the meaning behind being masculine in boxing is becoming less fixed and more open to a diverse conceptualization of masculinity.

#### 3.1.2. The Paradox of Looking Good

The traditional, hegemonic masculine identity was one storied as being encouraged by those in positions of power within the Canadian National Boxing Team. One boxer’s story highlighted how he felt he had to overcome one of his coach’s attitudes towards his appearance:

[Interviewer: Are there any parts of you that you feel not so comfortable sharing in your sport context?] Well when I had a little bit longer hair, I used to always gel my hair and stuff like that. I always made sure that my hair was gelled before my fights. And my coach he’d laugh at me. But it didn’t bother me… [Interviewer: I know you said it didn’t bother you at all. Did it ever feel like okay, just leave me alone, stop talking about me slicking back my hair?] Yeah. Because I know one coach, and he’s always making the same jokes, always. And it’s like, they get old. Like he’ll call me the princess (laugh). But it doesn’t bother me. I think that it’s like, man, your jokes get old. Like it’s not going to change.

(Ryan)

Ryan’s storying of styling one’s hair being viewed as a feminine trait by others, specifically using the effeminizing term “princess” by one of his coaches, provides a glimpse of the power coaches have over which identities are encouraged and/or discouraged through the words they use. While Ryan seems to have the confidence to brush off his coach’s jokes, he also speaks of having long hair and styling it in the past tense and laughs after telling the interviewer the name he is called. This could indicate that Ryan may have found it easier to give in to the expected norms; however, he finishes this part of his account with a defiant statement that things will not change. Further on in his story he provides further insight into why he chooses to take great care in what he looks like:

At my fight weight, or close to it, I feel better, feel lighter, faster, even stronger. … Well, when I’m close to my weight, I feel better because I look better. Yeah, to me I feel that I look better when I’m at weight. Look good, feel good, fight good (laugh). That’s what is said where it says “well-dressed” [in mandala]. I am always, you know, prepared like when I’m dressing for my fight, I am always making sure that stuff matches, you know, stuff like that… [Interviewer: What is the feeling around that? Like OK you’ve got the matching outfit; how does that affect you in your competition or your performance?] Well just if you look good, you feel good. That’s just my perspective. I just feel like I perform better when I’m well-dressed or whatever, like if I get my fresh hair cut (laugh).

(Ryan)

Ryan justified his behaviors as a way of boosting his confidence and power: “Look good, feel good, fight good.” He depicted his physical appearance as a reflection of his mental state, as when he looks good he feels well prepared. Again, the laugh at the end of the quote further insinuates Ryan’s realization that he goes against the expected behaviors of boxers held by those in position of power but has been able to move past this by developing his own understanding of masculinity. Answering whether he felt other boxers also cared about how they looked prior to entering the ring, Ryan mentioned how most men do consider their outfit and appearance before a fight, indicating that he did not feel alone in going against the expectations held by a figure of authority. Ryan’s view that he is not alone in caring about his appearance suggests that the expectations held by those in power may be outdated, as male boxers felt comfortable in expressing a desire to look good in order to feel optimally ready to perform at a high level.

### 3.2. Ethnicity Brings an Edge to Boxing

Ethnicity, the culture of people in a geographic region, including their language, heritage, religion, and customs [22], was mentioned by athletes as tremendously important to them, particularly for those not born in Canada. Through three subthemes, *Ethnicity brings differences and advancement*, *Religion as a source of empowerment*, and *Expressing identity through language* this section explores athletes’ accounts of how their ethnic identities and the manner in which they can express them has an impact on all aspects of their sport performance.

#### 3.2.1. Ethnicity Brings Differences and Advancement

The boxers described the boxing environment as one free from discrimination or deliberate separation, but this did not mean that ethnic differences were not apparent. Being born in a different country led to some natural differences for immigrant athletes:

I think a lot of them [teammates] notice that I’m more serious, like I’m more mature and more serious in the things that I do. They [teammates] respect that and they show that they understand. [Interviewer: Does that ever make you feel you are different, or not much part of the team, or an outsider because you have this different attitude?] No, not at all because I can get along with anyone. So, but that’s not bad for me. [Interviewer: Do you ever feel your teammates aren’t serious like they take things too, like a more relaxed approach, not really training like they should be?] No when we’re in training, when we’re in training camps they’re very serious because they’re training to win also. It’s just the way they think is different than the way I think because the way we’ve been raised and just different mentalities because of culture and religion and stuff like that ... It gives me an advantage because I listen properly, I don’t joke around and so in training there’s never a problem. In sparring, you know in training, I’m more aggressive. That makes me train harder.

(Oliver)

Oliver’s account of the ethnic and cultural basis for his higher level of commitment and different way of thinking begs us to consider how his identity construction occurs as an internalized process and one that is socially, culturally, and historically situated within each subculture that he is immersed. Oliver sees the differences between him and his teammates as a product of different cultural and religious upbringings (i.e., two identities). While he does not provide judgement about his teammates’ different approaches, he does believe that his serious approach has given him an advantage over others. Ethan’s account highlights how having a strong sense of ethnic identity can give athletes strength to draw upon in their performances:

My ethnicity means everything to me. It’s who I am, where I came from and how I grew up … My ethnicity is who I am and what could be my personality… I mean being [ethnicity] it means sort of, uh, strength. It’s a huge strength and I feel powerful in that sense. I wouldn’t have been the boxer that I am today if it wasn’t for my background. … they’re [people from his ethnicity] very strong people, they’re very mentally strong, they don’t break down. As a boxer, it helped me be strong in the brain and not backing down.

(Ethan)

Ethan’s narrative furthers the discussion on the role of an athlete’s ethnic and cultural background by providing a specific example of how these factors shape him as a fighter. Bounding his strength, both physical and mental, to his culture and ethnicity reveals a belief and pride in knowing where one is from and remaining loyal to his home culture. Loyalty to his home culture allows Ethan to feel confident when facing adversity, as he feels able to lean on the strength derived from his cultural upbringing. Thus, the consideration of one’s ethnicity and cultural values are presented as the foundation from which an athlete may build upon as they continue their journey in a host country.

#### 3.2.2. Religion as a Source of Empowerment

Freedom to practice one’s religion is an accepted right within boxing and as Ryan explained, is one that the boxers whose accounts are shared here wouldn’t give second thought to: “Definitely doesn’t get judged or nothing; nobody would get judged by that [religion]. Yeah, it’s definitely accepted.” Beyond mere acceptance, the dedication shown by certain athletes to their religion impressed those athletes for whom religion did not play a large role in their lives:

I think there’s [teammates] who are Muslim and that doesn’t bother me. Them they do their prayers during the day and it doesn’t bother me. I find, as a matter of fact, I find them assiduous, they always do their prayers.

(John)

The empowerment of religion within the boxing environment was portrayed as a source of strength and confidence directly prior to competition. Religious athletes derived strength from their ability to express themselves prior to entering the ring:

Let’s say I’m at a competition. I do my prayers, I do my things that, I’m supposed to do that makes me feel confident going into a fight and so on. I’ve done that [pray] at the Commonwealth Games I’ve done that at the Pan Am Games and it’s helped me a lot and helped me be confident with who I am in the ring.

(Ethan)

Ethan’s explanation of how he integrated prayers into his pre-performance routine highlights how feeling empowered to express aspects of one’s identity can be a powerful tool one can use to perform at one’s full potential. Feeling comfortable and secure in his freedom to express his religion allowed Ethan to feel confident and in control prior to performance. The acceptance of religion within boxing and the freedom to feel comfortable in praying also played a role in immigrant athletes feeling able to connect back with their home culture:

Yeah, I think religion is like a way home. Like I always knew it. If I fight or in my bedside I’m always praying, have time to pray. I would say I’m a religious person and I believe in God. So, it’s very important for me.

(Oliver)

Oliver’s description of religion as a way of finding home highlights the sociocultural basis for his connection to God and the important role that prayer plays in helping him feel connected with his home culture. The power of religion in Oliver’s life is rooted in his engagement with religion for as long as he can remember and is pervasive for him throughout his life, both in and out of sport. However, introduction to religious practices at an early age by one’s family did not mean that religion became an important aspect of every boxer’s identity: “I am not religious myself, to be honest. My family is, but I don’t worry about anything else other than boxing (laughter).” For Ryan, religion was presented as having a minimal impact on his life and sport performance, but his identification as non-religious indicates that the decision to not follow in his families chosen religion forms a part of his identity that still influences his life to some degree.

### 3.3. Expressing Identity through Language

Language as an element of one’s identity was present in three of the boxer’s stories, but stood out in Ryan’s mandala, with the words ‘English Speaking’ prominently displayed. This section of his identity was prominent due to the role it played in separating him from some of his teammates:

We’ll go to eat, and one half of the table will be the French [Francophone] guys and the other half will be the English [Anglophone] guys. It actually separated us. That’s what I noticed. Another thing was, we would be sitting down at the table and we would all be talking, and then they would be talking in French, and I would have no idea of what they were saying. I had no idea and I kind of felt left out. That’s how I used to feel. Now I don’t care. But I remember my first tournament I went on with the team last year, they were all speaking French at the table, and I was just sitting there like “man, they could be talking about me.” You know what I mean? That definitely separated me from them.

(Ryan)

The physical separation by the boxers into two camps, one Anglophone and the other Francophone, at meals highlights the power that communication barriers can have within a group. The lack of communication could harm the integration and unity of the team, showcased in this example by the boxer describing how he felt isolated when sitting with Francophone boxers and the fear he felt from them potentially talking about him without understanding if he was being made fun of. However, the last part of Ryan’s account indicates that this separation does not appear to be a fixed concept but one that is changing with his framing of this isolation as in the past. The continuation of this narrative indicates that this separation is bridged by the Francophone athletes trying to include the English-speaking boxer in their conversations by speaking English when he is with them:

On my last trip I just hung out with all the French guys … and even one of the guys said like “Alright, when [Ryan] is around we gotta try to speak English.” So like they’re trying, which I definitely appreciate it.

(Ryan)

The attempt to include their Anglophone teammate(s) indicates a willingness on the part of the Francophones to be inclusive, perhaps having not realized their exclusionary practices earlier on. Interestingly, Ryan’s account does not include a storyline in which he seeks help in learning French, but rather is more reliant on those around him conforming their behaviors to include him.

Language barriers were not solely presented as a team bonding problem, but also as having an impact on the support athletes could provide to each other during fights. Although primarily an individual sport, for some boxers the providing and receiving of support from teammates was a key to the way they described being a good teammate:

I felt like there was no point in calling out advice, because he’s not going to hear the English, he’s going to hear the French guys talking. So that kind of stopped me from, I don’t know, I don’t want to stop, because I want to cheer for him, I want to tell him what to do, but he’s not going to listen to me.

(Ryan)

The inability to provide this type of support was hard to swallow for Ryan whose description of this situation emphasizes the discomfort and marginalization an athlete can feel when faced with a division between teammates. The role of language in creating a divide is perhaps most evident here, as Ryan felt unable to fulfill his role as a teammate. The disconnect between himself and his teammate is presented as being a two-way process, with his portrayal of teammate as uninterested in his cheering due to language difference. Presenting this as a barrier that is not only for him to overcome may make this divide harder to cross than it needs to be, but without open discussion between teammates, these potentially misconceived notions may not be brought up to be resolved.

Interacting through bilingual teammates was one method presented by boxers as helping to bridge the gap between athletes identifying themselves solely as Anglophone and Francophone. For bilingual boxers, language did not play as large of a role in their identity within the boxing community, as their ability to speak both languages did not set them apart from any of their teammates and enabled them to negotiate power differently. John, a Francophone and bilingual boxer stated: “Yes, they are Anglophone, but that doesn’t bother me. I understand English well and I speak it well also.” Identifying as bilingual seemed to allow John to establish bonds with both Anglophone and Francophone boxers. John also helped to bridge the gap between other boxers, allowing for a stronger team unity to develop. Coaches were also presented as individuals that could be relied upon to speak both languages and therefore act as a bridge between teammates when the whole team was united for training camps or competitions. The importance of having someone able to bridge the gap between athletes who spoke different languages was fully recognized by the national team coaches: “He’ll say it [instruction] first in English, and then translate it to French for the other guys. Or he’ll do it the other way around, where he’ll tell them first and then tell us. But yeah, he usually does both.” Ryan’s description of the coach’s ability to speak both English and French as allowing him to feel supported as an Anglophone, while portraying of Francophone athletes as the “other”, further solidifies his identification as an Anglophone. This suggests that, despite the coach’s best efforts, there is still more to be done until the boxer begins to identify himself as part of the team rather than solely as an Anglophone.

## 4. Discussion

Our aim within this submission was to bring a more focused lens to the identities that members of the Canadian National Men’s Boxing Team felt were relevant to their stories of performance and how accepted they felt these identities were within the boxing context. The four boxer’s stories presented here reveal major storylines associated with their identities in relation to their gender (i.e., male), ethnicity, religion, and language (i.e., Francophone, Anglophone, bilingual). The way in which the accounts are told highlight their description of the “preferred” identities in their social context, the expected behaviors that go along with these preferred identities, and the way in which they differ or conform to these identities [2]. Although our analysis is told through snapshots of each identity, as opposed to previous work on the intersection of identities, a careful reading reiterates conclusions from other researchers that athletes’ identities constitute a dynamic and multi-layered construct formed by personal and social processes in and outside of sport [6,7].

Masculinity is presented by boxers as being an identity that carries with it certain expectations that are encouraged and discouraged by those in positions of power within the boxing context. While one boxer explained it benefits boxers to fit the hegemonic notion of masculinity empowered within boxing, another brings to light his feeling that this narrow notion of masculinity is one that does not work well for him. Ryan’s explanation of his deviation from the expected norm reveals an athlete who feels at odds with coaches who he expects to receive support from. His resistance to the dominant narrative is similar to that of “Alex”, an Olympic medalist who did not follow the mandated performance narrative expected of him, and “Joe”, an Australian football league (AFL) player who attempted to resist the mental toughness ideals encouraged within his sport [2,23]. Ryan’s account presented here rings similar to Alex, in that he speaks about finding his own path to success, regardless of the opinions of those in power; however, his actions suggest he is more similar to Joe, an athlete who adapted his story in order to become accepted into his sporting subculture. While Ryan maintains that he does not care about his coach’s jokes, the resistant actions (i.e., longer hair and the use of gel) are presented as actions of the past, suggesting that he has perhaps conformed to the masculine behaviors encouraged by his coach. His story indicates that athletes may move fluidly through the three distinct identity negotiation processes proposed by Carless and Douglas [2]. While Ryan speaks about resisting the dominant narrative of an athlete within his sport, his actions perhaps indicate that he has begun to live the part of an athlete to avoid being different to those around him. His decision to stop resisting the dominant narrative is one that may be troubling for applied practitioners. Schinke and colleagues on behalf of the International Society of Sport Psychology warn that an athlete’s decision to conform to the dominant narrative may potentially be damaging to their wellbeing and development [1]. Ryan’s story does not suggest this to be the case at the time of his storytelling, but the snowballing of this decision may become problematic. Additionally, Ryan and Oliver both extol the role that their burgeoning talent and success as a boxer has on their ability to resist the dominant narrative. Their stories highlight a troubling aspect of elite sport, the reliance on performance to define who they are. Following on Carless and Douglas’ recommendations, there is a need for those in power to reflect on the stories that are promoted within sport and to encourage the sharing of alternatives [2]. Going beyond this, it is also incumbent on those in power to recognize that athlete’s identities are constantly in a state of flux and that the need to share stories is a constant one rather than a one-time experience.

The ethnic and religious narratives of these boxers’ stories in relation to their identity revealed a much more open and accepting context than for gender. This may stem from work conducted by the fourth author in helping develop cultural competence on the part of the team’s coaches and create space within this boxing subculture for different ethnic and religious stories [14]. Embracing the culturally diverse nature of the national boxing team, a focus of his work became developing cross-cultural communication among athletes and coaches. The result of this work can be seen in the stories told by other immigrant athletes on the national boxing team who have described their feelings of pride in being able to represent Canada on the world stage [24]. However, the journey towards representing Canada is one filled with twists and turns and the stories told by the boxers in this study reveal a novel way in how they deal with times of difficulty during the journey. The two immigrant boxers’ accounts showcased here bring to light the influential nature of their culture of origin and how they felt stronger and more supported due to being able to connect back to their roots, while also being proud to represent Canada. Berry would describe these boxers as having positively integrated into Canadian society due to their ability to feel connected with Canadian culture while retaining their home culture connection [25]. The boxers’ accounts not only showcase a feeling of being integrated into the Canadian boxing team, but also highlight the influential role their home culture plays as a source of confidence for these boxers. Feeling a sense of pride in their home cultures is seen by these boxers as an advantage that they have over other boxers, as they present their differences in a positive light.

Similarly, the boxers’ accounts portrayed here reveal that both religious and non-religious athletes feel encouraged to approach (or not approach) religion in their own way. Religious athletes’ stories revealed a sense of acceptance within the boxing environment, portrayed by how they spoke of feeling comfortable in praying before fights. The use of prayer prior to fights has previously been promoted as a method to help enhance performance and well-being before, during, and after competition [26]. This may be due to allowing athletes to feel more confident prior to performance and giving athletes a reflective time following competition during which deeper meaning can be given to their success or failure [27]. For boxers, competition is not only outcome dependent but also carries the ever-present risk of injury and/or inability to meet one’s own personal expectations. For athletes for whom prayer is a part of who they are and not just a pre-performance ritual, prayer can provide them with a powerful connection to a greater being and help reduce the anxiety that comes along with uncertainty, allowing them to focus on their performance [28].

The storying of language as an integral component of one’s identity by one boxer presents an interesting insight into the way identities are shaped and constrained within an individual’s macro- and micro-level sociohistorical contexts [29]. The tension felt by Ryan due to his inability to speak the same language as some of his teammates reflects the prominent role that language plays in allowing an individual to shape who they are through their language and discourse [30]. On the surface the Canadian National Boxing Team reflects the larger Canadian social context, in that it is a multi-lingual context comprised of athletes whose mother tongue is English, French, or another native language. Understanding the historical context of bilingualism in Canada allows for a deeper understanding of the tension that exists between English and French speakers. Despite being a bilingual country, English and French have never been equal languages in Canada, with French being confined to very few regions outside of its main base in the province of Quebec. With increased immigration to Canada, the presence of French speaking regions has further declined as many immigrants have chosen to adopt English rather than French [31]. This may be due in part to English becoming the global language, spreading across the world as the official language in many countries and the most widely taught second language [32]. The perceived dominance of the English language is evident in Ryan’s account as he tells about how his teammates were tasked with speaking their second language to accommodate his inability to speak French. Though Ryan’s Francophone teammates willingly complied most of the time, there remained times of discord when they reverted to French, particularly during stressful times such as competition. Ryan did not express a need to learn French, but rather seemed to prefer subverting his identity due to being unable to express himself as he wished he could. The reluctance to adapt may be akin to members of a dominant culture tasking immigrants with the responsibility to change when they arrive in a new society rather than being willing to engage in a shared process of change (i.e., acculturation loads) [33]. Ryan finds himself at odds, as outside of the boxing context he may feel he is part of the dominant culture; however, within the boxing context Ryan’s story resembles the alienation felt by immigrants tasked with shouldering the need to change. Although the roles are switched, with Ryan believing the responsibility to change lies with his teammates, his feeling of alienation is due to his reluctance to engage in a reciprocal relationship of learning. Feeling alienated resulted in Ryan feeling unable to express himself as he wished he could, leaving him unable to perform his identity.

The identities presented in this paper do not comprise the full range of those expressed by athletes through their stories. The identities included, however, were discussed in depth, allowing us to provide a clear and critical storyline. This is not to suggest that these identities deserve more attention than others, it is merely the practical approach taken by us in our analysis of these four stories. Future researchers should look to provide a more extensive overview of the range of identities presented by athletes, rather than constraining stories to those that are “more interesting.” As researchers and practitioners, a goal should be to critically look at the stories we choose to tell and those we dismiss and why this is the case, as this may lead to the perpetuation of dominant storylines over others.

## 5. Conclusions

The present article responds to calls for more critical examinations of the marginalization of athletes’ identities due to a dominant performance narrative within the realm of sport. The findings presented showcase a boxing environment that is becoming increasingly inclusive of identities that do not fit the dominant historical narratives. Contrasting stories told by the boxers identifying with different narratives of masculinity, ethnicity, religion, and language reveal an environment that is more inclusive of some differing narratives to others. The discouragement of alternative identities in relation to masculinity exposes the potential negative impact that uncritically reproduced narratives can have on individuals. However, the space afforded alternative ethnic, religious, and language identities can be attributed to the willingness of individuals in power to recognize the need to allow for cultural communication to become a two-way process. The acceptance and encouragement of an open approach to differences on the part of those in power reveals an understanding of the positive influence on performance that empowering individuals to “be themselves” can have. Importantly, successful performance is not the only positive by-product of an empowering context, improved mental health may also result. The ability for athletes to feel comfortable in feeling empowered to express their idiosyncratic identities has been found to result in a healthy training and competition environment. Additionally, the role of language is showcased as a construct of an athlete’s identity that helps empower or disempower other identities. The inability to speak with those around them limits an individual in his ability to perform the behaviors associated with his identities (i.e., as a supportive teammate) and can leave athletes feeling frustrated and withdrawn. Helping athletes develop methods of communication would surely aid in the development of a more inclusive context.
